# Supplementary material for: Emergence of belief-like representations through reinforcement learning
Source: bioRxiv. 2023 Apr 4:2023.04.04.535512. Preprint. [Version 1] doi: 10.1101/2023.04.04.535512 (PMC10104054; doi:10.1101/2023.04.04.535512)
Supplement: Supplement 1 [file NIHPP2023.04.04.535512v1-supplement-1.pdf]

bioRxiv preprint doi: https://doi.org/10.1101/2023.04.04.535512; this version posted April 7, 2023. The copyright holder for this preprint (which was not certified by peer review) is the author/funder, who has granted bioRxiv a license to display the preprint in perpetuity. It is made available under aCC-BY 4.0 International license.

Supplemental Figures

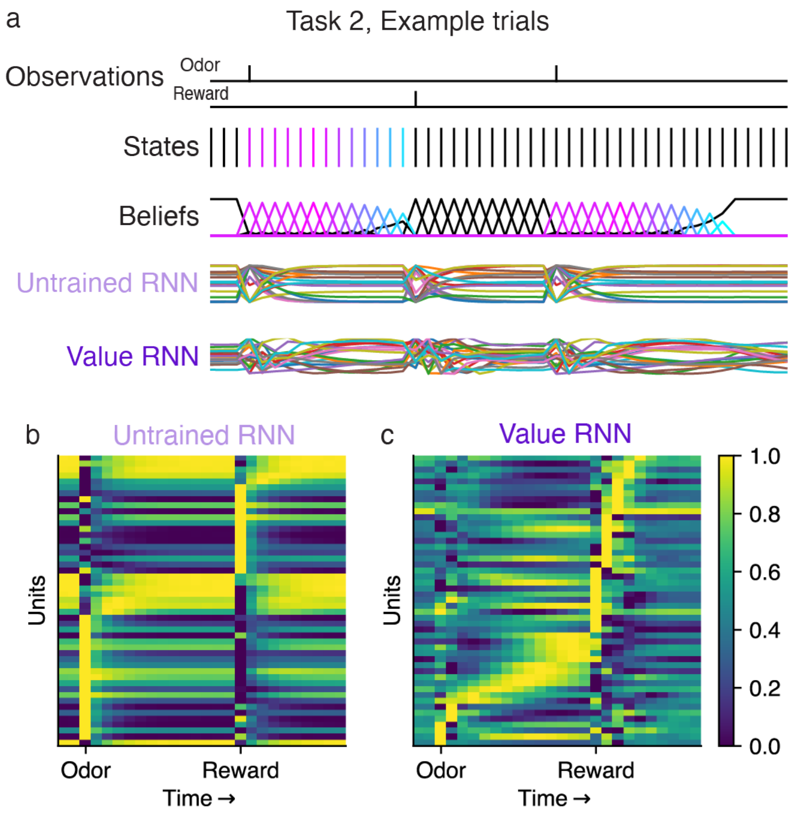

Fig S1. RNN activity before and after training on Starkweather Task 2. a. Observations, states, beliefs, and RNN activations on two example trials from Task 2. **b-c.** RNN unit activity (individually normalized to span between 0 and 1), with units sorted by time of peak activation on held-out trials, on an RNN before (panel **b**) and after (panel **c**) training. Both before and after after training, RNN units exhibited tuning to elapsed time following observations, with variance that scaled with elapsed time.

bioRxiv preprint doi: https://doi.org/10.1101/2023.04.04.535512; this version posted April 7, 2023. The copyright holder for this preprint (which was not certified by peer review) is the author/funder, who has granted bioRxiv a license to display the preprint in perpetuity. It is made available under aCC-BY 4.0 International license.

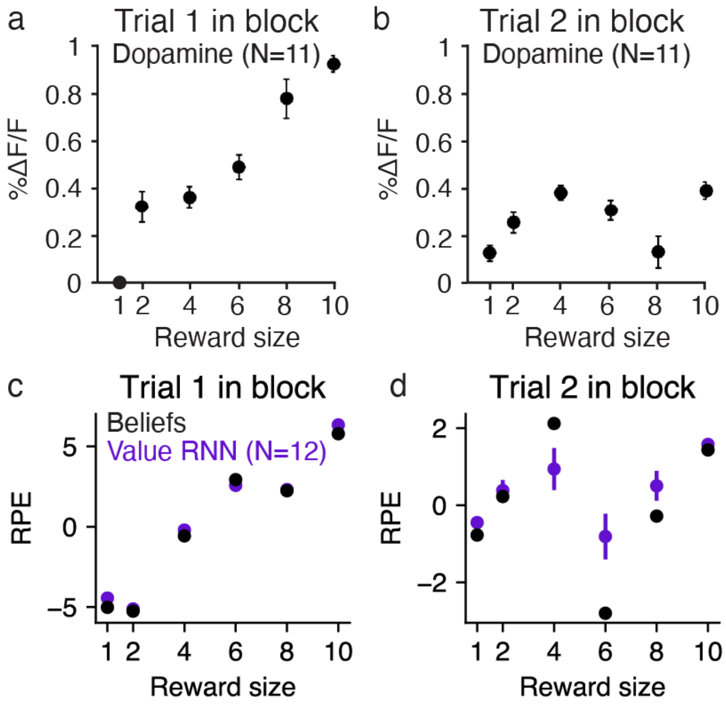

Fig S2. Value RNNs trained on the Babayan task recapitulate dopamine activity and belief RPEs in response to intermediate reward sizes. a-b. Average dopamine response on trial 1 (panel **a**) and trial 2 (panel **b**) during probe sessions including blocks with intermediate reward sizes. Circles and lines depict mean ± SE across $N = 11$ animals. Reproduced from Babayan et al. [10]. **c-d.** Same as panels **a-b**, but for the RPEs of the Belief model (black) and Value RNNs (purple). Value RNNs were trained on sessions including only blocks with rewards $r_t \in \{1, 10\}$, as in the main text. Value weights for the Belief model and Value RNNs were fit using a test session including 39 blocks each with $r_t = 1$ and $r_t = 10$, and 3 blocks each with $r_t \in \{2, 4, 6, 8\}$, similar to the proportions used in Babayan et al. [10]. RPEs were then measured on a different test session. Purple circles and lines depict mean ± SE across $N = 12$ models.

bioRxiv preprint doi: https://doi.org/10.1101/2023.04.04.535512; this version posted April 7, 2023. The copyright holder for this preprint (which was not certified by peer review) is the author/funder, who has granted bioRxiv a license to display the preprint in perpetuity. It is made available under aCC-BY 4.0 International license.

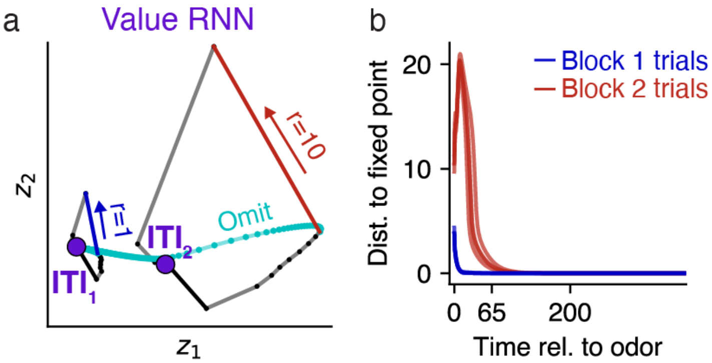

Fig S3. Value RNNs trained on the Babayan task exhibit one fixed point. a. RNN activity during two example trials, one during Block 1 (left) and the other during Block 2 (right). Same as Fig 7D. Here we also include RNN activity trajectories if each reward had been omitted. While activity for the Block 2 trial initially returns to the putative ITI_2 state, it eventually returns to the true fixed point at ITI_1 **b.** Distance of RNN activity from the single fixed point (e.g., ITI_1 in panel **a**) following an odor observation (i.e., an omission trial). While the maximum ITI duration is theoretically infinite, the maximum ITI duration in the training data was at $t = 65$. RNN activity on Block 2 trials therefore remained separate from the activity on Block 1 trials for the range of experienced ITI durations.
